# Perioperative Acute Kidney Injury and Prognosis of Infants With Down Syndrome and Congenital Heart Disease

**DOI:** 10.7759/cureus.74658

**Published:** 2024-11-28

**Authors:** Tsubasa Shimozono, Kentaro Ueno, Eri Okuda, Yoshihiro Takahashi, Koji Nakae, Junpei Kawamura, Yasuhiro Okamoto

**Affiliations:** 1 Pediatrics, Kagoshima University Hospital, Kagoshima, JPN

**Keywords:** acute kidney injury, cardiac surgery, congenital heart disease, down syndrome, hypoplastic kidney, renal dysfunction, urinary tract malformation

## Abstract

Background

Children with Down syndrome (DS) often have hypoplastic kidneys and urinary tract malformations that increase their renal dysfunction risk. They also have a higher congenital heart disease (CHD) rate, requiring cardiac surgery during infancy. Renal dysfunction in such patients may be associated with the development of cardiac surgery-associated acute kidney injury (CS-AKI), but this remains unclear. In this study, we compared the incidence, severity, and prognosis of CS-AKI between infants with and without DS complicated by CHD.

Methods

We retrospectively analyzed 144 consecutive infants with (n=59) and without (n=85) DS who underwent cardiac surgery for CHD between January 2013 and October 2018. The primary endpoint was CS-AKI incidence, and the secondary endpoints were CS-AKI severity and perioperative prognosis. We assessed the severity of CS-AKI using the Kidney Disease: Improving Global Outcomes (KDIGO) criteria.

Results

The DS group had significantly smaller kidney size when measured by ultrasound at birth than the non-DS group (*P*<0.001). Preoperative renal function assessment revealed significantly higher serum creatinine (Cr, 0.29 vs. 0.20 mg/dL, *P*<0.001) and lower Cr-estimated glomerular filtration rates (82.0 vs. 101.4 mL/min/1.73 m^2^, *P*<0.001) in the DS group than in the non-DS group. CS-AKI incidence and severity did not differ between the groups. Risk factors for CS-AKI incidence included being younger at the time of cardiac surgery and a prolonged cardiopulmonary bypass in the overall cohort and DS group.

Conclusions

The incidence and severity of CS-AKI did not differ between the DS and non-DS groups. Understanding potential renal dysfunction and managing patients with DS and CHD may assist in preventing perioperative acute kidney injury after cardiac surgery.

## Introduction

Congenital heart disease (CHD) significantly contributes to morbidity and mortality in children with Down syndrome (DS), affecting up to 50% of this population [1]. Children with DS have improved survival outcomes through birth plans supported by specialists, referral to expert centers for prenatal and neonatal diagnosis, and early CHD repair for infants eligible for biventricular repair. However, prognostic factors remain challenging, including cases with single ventricular disease, perioperative complications such as chylothorax and pericardial effusion, pulmonary hypertension, upper airway obstructive disorders, issues specific to DS such as obesity and metabolic disorders, as well as the challenges of cardiac care for patients with DS in low- and middle-income countries [1,2]. Additionally, children with DS are known to have significantly smaller kidneys and a higher prevalence of renal and urinary tract abnormalities compared to age-matched control groups [3].

In patients with CHD, cardiac surgery-associated acute kidney injury (CS-AKI) complicates approximately 30-40% of cases and can be life-threatening [4]. Meta-analyses have reported risk factors associated with CS-AKI development in pediatric cardiac surgery, including younger age, prolonged cardiopulmonary bypass time, perioperative fluid overload, pulmonary hypertension, and use of vasoactive agents [4]. Children with DS frequently present with CHD, necessitating corrective cardiac surgery in infancy [1], when renal function remains immature. However, the impact of renal function on the development of perioperative CS-AKI and mortality rates in DS children remains unclear.

Therefore, this study investigated the incidence, severity, and prognosis of CS-AKI during infancy in patients with DS complicated with CHD, comparing these outcomes with those observed in patients without DS.

## Materials and methods

The Kagoshima University Ethics Committee approved the study (approval number 190055) in accordance with the International Conference on Harmonization Good Clinical Practice Guidelines and the Declaration of Helsinki. The need for informed consent from guardians and/or patients was waived because of the retrospective nature of the study. A total of 263 infants who underwent cardiac surgery between January 2013 and October 2018 were included in the study. We evaluated infants aged 3 months to 2 years, a period characterized by insufficient renal function compared to adults, allowing for the estimation of glomerular filtration rate (e-GFR; mL/min/1.73 m2) from serum creatinine (Cr) levels (mg/dL). Infants who were premature, or had severe neonatal asphyxia, chromosomal, brain, or genetic abnormalities (other than DS), coagulation disorders, or missing data were excluded from the study. Since most surgical procedures for CHD associated with DS consist of biventricular repair, including ventricular and atrioventricular septal defects as well as tetralogy of Fallot, we excluded infants with single ventricular disease.

**Figure 1:**
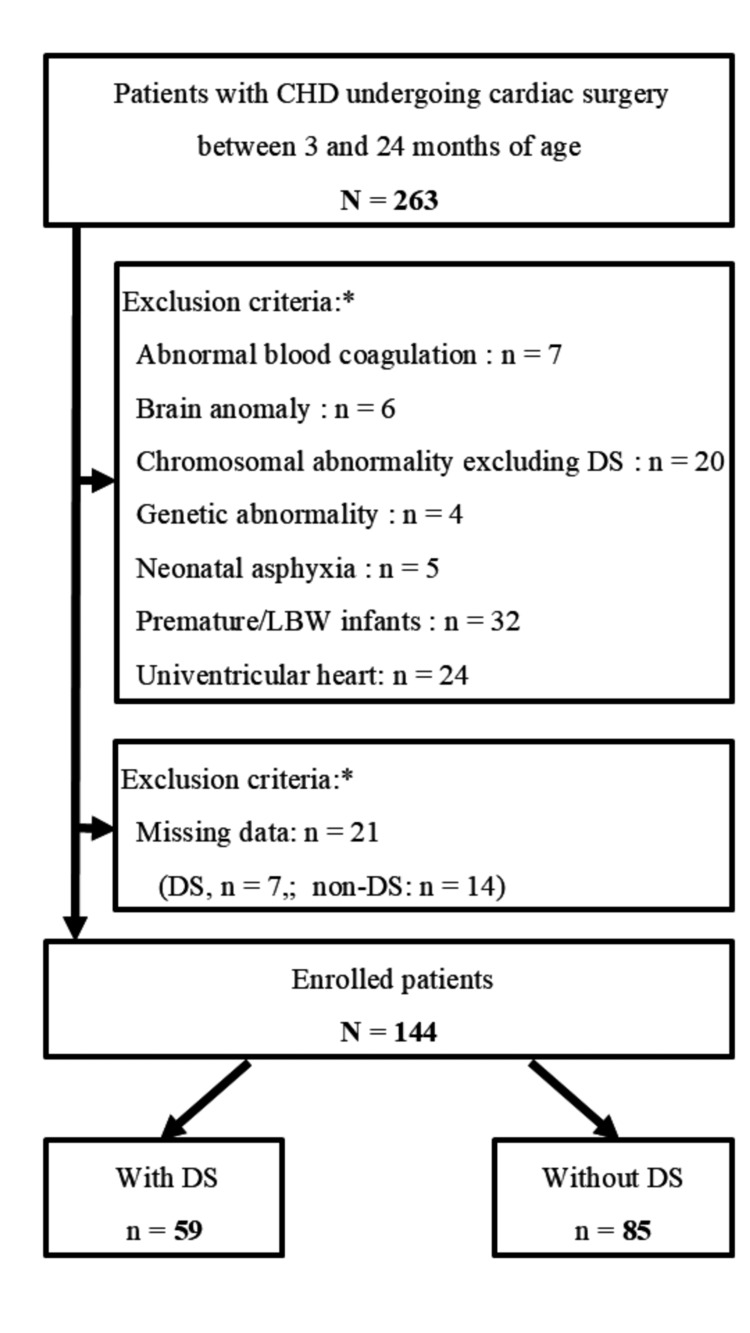
Flow chart of study identification, inclusion, and exclusion criteria. The study included 263 patients aged 3 months to 2 years who underwent cardiac surgeries during the study period. A total of 119 patients were excluded from the analysis for the following reasons: chromosomal abnormality excluding Down syndrome (n=14), genetic abnormality (n=4), neonatal asphyxia (n=5), premature/low birth weight infants (n=32), brain abnormality (n=6), blood coagulation abnormality (n=7), and univentricular heart (n=24). Finally, 144 patients were included in the analysis. Enrolled patients were divided into two groups: DS (n=59) and non-DS (n=85). * The exclusion criteria are divided by the primary disease to avoid duplication of patients. Abbreviations: CHD, congenital heart disease; DS, Down syndrome; LBW, low birth weight

Ultimately, 144 patients with CHD (59 with DS and 85 non-DS) were enrolled and retrospectively analyzed (Figure 1).

We retrospectively investigated factors such as the diagnosis of CHD, age at surgery, sex, length of the long axis of kidneys (mm), and ratios of the patients’ kidney size compared with normal kidney size (%N). Kidney length was measured by two neonatologists, and the average value was used after confirming no significant differences between their measurements. In addition, kidney size (%N) was calculated using the proposed formula “Height (m) x 5 + 2” based on the average kidney length (cm) of Japanese children on ultrasound examination [5]. We also analyzed the use of medications before cardiac surgery, weight of the children at the time of surgery, preoperative serum Cr (sCr) levels, Cr-based eGFR (Cr-eGFR), presence of preoperative cyanosis, comorbidities (pulmonary hypertension, upper airway obstructive disease, and gastrointestinal disease), perioperative factors such as cardiopulmonary bypass time, and maximum vasoactive-inotropic score within 48 h after cardiac surgery (i.e., dopamine dose (μg/kg/min) + dobutamine dose (μg/kg/min) + 100 × epinephrine dose (μg/kg/min) + 10 × milrinone dose (μg/kg/min) + 25 × olprinone dose (μg/kg/min) + 100 × norepinephrine dose (μg/kg/min)) [6]. Regarding Cr-eGFR, we employed a calculation formula applicable to Japanese children aged 3 months to 2 years [7]:

Reference sCr levels (ref Cr) were determined using equations based on body length (x), separately for males and females. For males: ref Cr = −1.259x 5 + 7.815x 4 − 18.57x 3 + 21.39x 2 − 11.71x + 2.628. For females: ref Cr = −4.536x 5 + 27.16x 4 − 63.47x 3 + 72.43x 2 − 40.06x + 8.778

Provisional GFR = 110.2 × (ref Cr/patient’s sCr) + 2.93

R = 0.107 × ln (age; months) + 0.656

eGFR = provisional GFR × R

For CS-AKI, we assessed the severity using the Kidney Disease: Improving Global Outcomes (KDIGO) classification system [8]. According to KDIGO criteria, CS-AKI severity is categorized as follows: Stage 0: No change or an increase in sCr of less than 0.3 mg/dL, with urine output exceeding 0.5 mL/kg/h. Stage 1: An increase in sCr of 0.3 mg/dL or more within 48 h or an increase of 1.5-1.9 times the baseline value, with urinary output less than 0.5 mL/kg/h within 6-12 h. Stage 2: An increase in sCr by 2-2.9 times the baseline value, with urinary output less than 0.5 mL/kg/h for more than 12 h. Stage 3: An increase in sCr by 3 times the baseline value or reaching 2.5 mg/dL or more, initiation of renal replacement therapy, urinary output of less than 0.3 mL/kg/h for more than 24 h, or anuria for more than 12 h (the baseline value refers to the sCr value before the onset of acute kidney injury (AKI)). Moreover, upper airway obstruction was defined as the need for airway support devices or visualization of obstructed upper airway images via fiber optic endoscopy before cardiac surgery.

In this study, the primary endpoint was the incidence of CS-AKI in both the DS and non-DS groups. Secondary endpoints included risk factors for CS-AKI, postoperative outcomes such as the need for renal replacement therapy (RRT), intensive care unit (ICU) stays, and in-hospital mortality within 14 days.

Statistical analysis

Continuous variables were presented as medians with interquartile ranges (IQRs; 25th-75th percentile), whereas categorical variables were expressed as frequencies and percentages. Kidney size was measured by two neonatologists using in-computer renal echocardiographic images to measure the length of the long axis of kidneys, and Fleiss' Kappa statistics were used to assess interobserver variability. Baseline comparisons between the DS and non-DS groups were conducted using the Mann-Whitney U test for continuous variables and chi-square analysis for categorical variables. Risk factors were determined using multivariate logistic regression analysis and multiple regression analysis. To identify predictor factors, the multicollinearity of all predictor factors was initially assessed using the criterion of variance inflation factor (VIF) >10 and then factors with VIF <10 entered into the model using logistic regression analysis and multiple regression analysis. Statistical significance was set at P < 0.05. All statistical analyses were performed using the Statistical Package for the Social Sciences (SPSS) for Windows, version 25.0 (IBM Corp., Armonk, USA).

## Results

Comparison of DS and non-DS groups and the incidence of CS-AKI

All children with DS had standard trisomy 21, with ventricular septal defect in 36 (61.0%), atrioventricular septal defect in eight (13.6%), and tetralogy of Fallot in eight (13.6%) cases. Gestational age did not differ between the DS and non-DS groups; however, the DS group had smaller birth height and weight as well as a smaller renal length (mm) (39 vs. 42, P<0.001) (Fleiss' Kappa, 0.872; 95% confidence interval, 0.823-0.908, P<0.001) and kidney size (%N) evaluated by ultrasound compared to the non-DS group (86.3% vs. 93.0%, P<0.001). Preoperative complications were more frequent in the DS group than non-DS group, with a higher incidence of pulmonary hypertension (46 (78.0%) vs. 26 (30.6%), P<0.001), upper airway obstruction disease (14 (23.7%) vs. 6 (7.1%), P=0.004), and gastrointestinal disease (3 (5.4%) vs. 0, P=0.036).

Regarding perioperative backgrounds, no difference existed in the use of diuretics or angiotensin-converting enzyme (ACE) inhibitors, surgical timing, and preoperative height/weight between the DS and non-DS groups. In preoperative renal function evaluation, the DS group had significantly higher sCr levels compared to the non-DS group (0.29 (0.27-0.34) vs. 0.23 (0.20-0.28), P<0.001), and Cr-eGFR was significantly lower in the DS group (82.0 (73.3-91.9) vs. 101.4 (86.3-115.0), P<0.001). No difference was observed in surgical duration between the DS and non-DS groups. Postoperative data showed no difference in the use of vasoactive-inotropic score and infection between the DS and non-DS groups, but the DS group had less fluid volume than the non-DS group, as assessed by in-out balance. The incidence of CS-AKI was 38 cases (64.4%) in the DS group and 59 (69.4%) in the non-DS group. Seven cases (11.9%) were severe (KDIGO 3) in the DS group and 14 (16.5%) in the non-DS group, with no significant difference in severity between the two groups (Table 1).

**Table 1:** Comparison of clinical characteristics, cardiac catheterization data, and renal function between infants with and without (control) Down syndrome. Data are expressed as medians with interquartile ranges (ICRs; 25th‒75th percentiles) or as numbers (n) with proportions (%). Baseline comparisons between the DS and non-DS groups were conducted using the Mann-Whitney U test for continuous variables and chi-square analysis for categorical variables. * A small number of patients had overlapping surgical site infection and bacteremia. ACE, angiotensin converting enzyme; AKI, acute kidney injury; ASD, atrial septal defect; AVSD, atrioventricular septal defect; CHD, congenital heart disease; CPB, cardiopulmonary bypass; Cr, creatinine; DS, Down syndrome; eGFR, estimated glomerular filtration rate; ICR, intracardiac repair; ICU, intensive care unit; KDIGO, Kidney Disease: Improving Global Outcomes; NS, not significant; TOF, tetralogy of Fallot; VSD, ventricular septal defect.

Characteristics	DS (n = 59)	Control (n = 85)	P
Patient's birth history			
Gestational age	38.3 (37.0‒38.9)	38.6 (36.8‒39.2)	0.908
Sex (female)	22 (37.3%)	46 (54.1%)	0.047
Height (cm) at birth	49.4 (48.8‒50.6)	50.2 (49.4‒51.4)	0.003
Body weight (kg) at birth	2.9 (2.8‒3.0)	3.0 (2.9‒3.2)	< 0.001
The long axis of kidney size (mm) at birth	39 (38‒40)	42 (41‒43)	< 0.001
Long axis of kidney at birth (% normal)	86.3 (83.7‒89.0)	93.0 (90.3‒98.7)	< 0.001
Complications			
Cyanosis	10 (16.9%)	12 (14.1%)	0.643
Upper airway obstruction	14 (23.7%)	6 (7.1%)	0.004
Pulmonary hypertension	46 (78.0%)	26 (30.6%)	< 0.001
Gastrointestinal diseases	3 (5.4%)	0	0.036
Diagnosis of CHD			
VSD	36 (61.0%)	64 (75.3%)	0.067
AVSD	8 (13.6%)	3 (3.5%)	0.056
TOF	8 (13.6%)	8 (9.4%)	0.611
ASD	3 (5.1%)	2 (2.4%)	0.676
Others	4 (6.8%)	8 (9.4%)	0.798
Medications before cardiac surgery			
Diuretics	45 (76.3%)	72 (84.7%)	0.202
ACE inhibitors	8 (13.6%)	19 (22.3%)	0.266
At time of ICR			
Age (months)	6.0 (4.0‒11.0)	5.5 (3.9‒8.2)	0.331
Height (cm)	61.8 (56.7‒69.4)	60.9 (57.9‒66.8)	0.898
Body weight (kg)	5.4 (4.5‒7.3)	5.4 (4.5‒7.0)	0.982
Cr (mg/dL)	0.29 (0.27‒0.34)	0.20 (0.23‒0.28)	< 0.001
Cr‒eGFR (mL/min/1.73 m^2^)	82.0 (73.3‒91.9)	101.4 (86.3‒115.0)	< 0.001
Surgical data			
CPB duration (min)	115.0 (85.5‒147.5)	111.0 (91.0‒142.0)	0.997
Cross‒clamp duration (min)	69.0 (46.5‒87.5)	68.0 (53.0‒93.0)	0.582
Postoperative data			
Vasoactive‒inotropic score	12.0 (10.0‒14.0)	10.8 (7.4‒13.5)	0.280
Fluid (mL/kg/day)	45.0 (44.0‒47.5)	48.0 (45.0‒50.0)	< 0.001
Infection (total)	6* (10.2%)	9* (10.6%)	0.936
Surgical site infection	3 (5.1%)	4 (4.7%)	N.S.
Bacteremia	4 (6.8%)	4 (4.7%)	0.869
Ventilator-associated pneumonia	2 (3.4%)	3 (3.5%)	N.S.
AKI	38 (64.4%)	59 (69.4%)	0.529
KDIGO 1	18 (30.5%)	25 (29.4%)	0.629
KDIGO 2	13 (22.0%)	20 (23.5%)	0.974
KDIGO 3	7 (11.9%)	14 (16.5%)	0.714
Postoperative outcomes			
Renal replacement therapy	1 (1.7%)	2 (2.4%)	N.S.
ICU stay (days)	4.0 (3.0‒6.0)	2.0 (2.0‒4.0)	< 0.001
In-hospital mortality	0	0	N.S.

Risk factors for CS-AKI and perioperative prognosis between the DS group and non-DS group

Risk factors for CS-AKI development, when combining the DS and non-DS groups, included younger age at the time of surgery and longer cardiopulmonary bypass time (P=0.001, odds ratio (OR) 0.818, 95% confidence interval (CI) 0.725-0.921; P<0.001, OR 1.019, 95% CI 1.010-1.028). Regarding perioperative prognosis, no deaths occurred during this period and there was no difference in RRT between the DS and non-DS groups; however, the intensive care unit (ICU) stay was prolonged in the DS group compared to the non-DS group. Concerning renal function and CS-AKI in the DS group, risk factors for perioperative CS-AKI development in children with DS mirrored those in the entire cohort, with younger age at the time of surgery and longer cardiopulmonary bypass time being significant risk factors (P=0.014, OR 0.828, 95% CI 0.704-0.961; P=0.002, OR 1.017, 95% CI 1.006-1.029).

**Figure 2:**
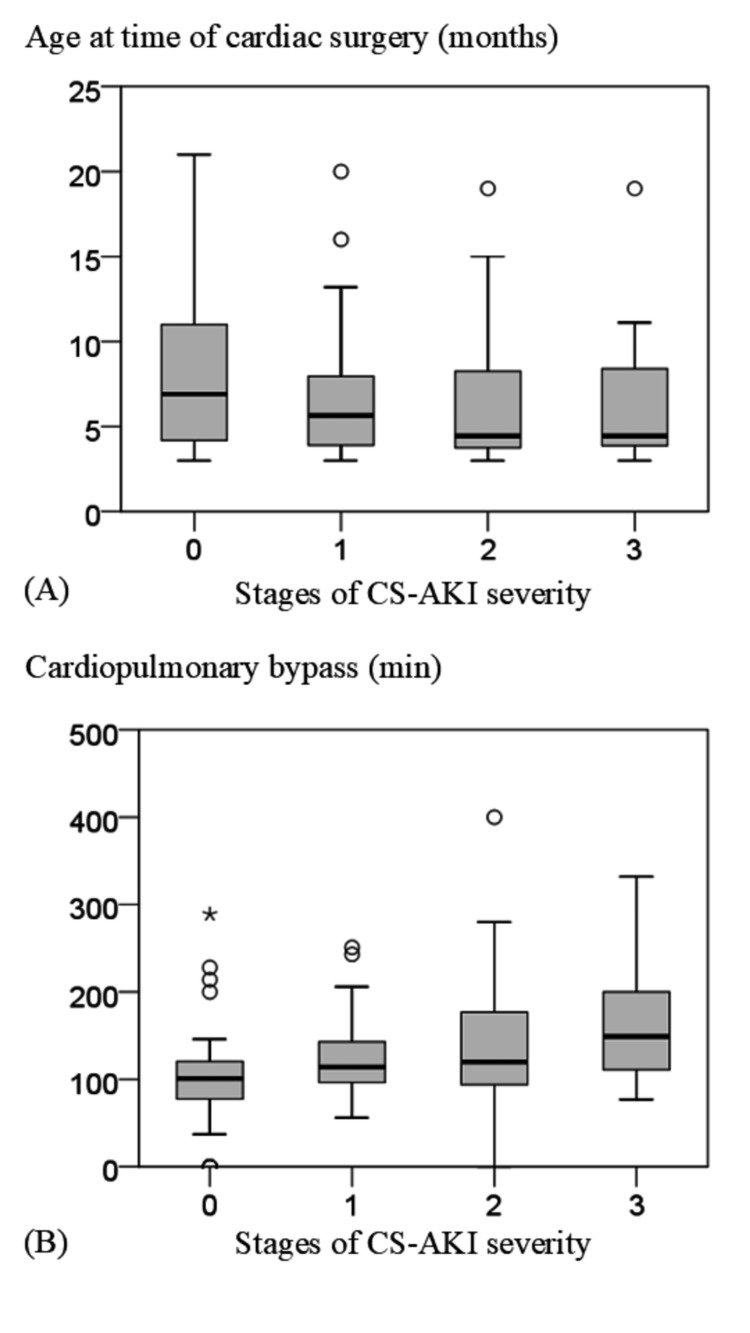
Risk factors based on KDIGO stages for CS-AKI severity. Risk factors related to the severity of CS-AKI in the KDIGO classification showed that cases with higher severity had a younger age at the time of surgery and longer cardiopulmonary bypass time (P=0.007 and P<0.001) in multivariate logistic regression analysis. (A) Age at cardiac surgery and staging of CS-AKI severity. (B) Cardiopulmonary bypass time and staging of CS-AKI severity. The asterisk (*) in the figure indicates outliers. Abbreviations: CS-AKI, cardiac surgery-associated acute kidney failure; KDIGO, Kidney Disease: Improving Global Outcomes.

Factors related to the severity of CS-AKI in the KDIGO classification showed that cases with higher severity had a younger age at the time of surgery and longer cardiopulmonary bypass time (P=0.007 and P<0.001, respectively; Figure 2). In multiple regression analysis, the presence of preoperative airway obstruction disorders was identified as a contributing factor to longer ICU stay (R=0.552, P<0.001).

## Discussion

Children with DS have a higher rate of anatomical abnormalities compared to healthy children, and they also have smaller kidney sizes and fewer nephrons due to hypoplastic kidneys [3], suggesting potential renal dysfunction that may affect long-term renal outcomes. Patients with DS have smaller kidneys and a high incidence of congenital kidney and urinary tract anomalies including histological immaturity and morphologically abnormal glomeruli, suggesting impaired nephrogenesis and glomerular development [3]. Therefore, this study examined renal function in children with DS undergoing surgery in infancy due to CHD to determine its association with perioperative CS-AKI and ICU management.

Our study revealed the following: 1) The incidence of CS-AKI was 67.4% (64.4% in the DS group and 69.4% in the non-DS group), with no differences in CS-AKI severity based on the KDIGO classification between the groups; 2) risk factors for CS-AKI development in the DS group and the overall cohort (DS and non-DS groups) included a younger age at the time of cardiac surgery and longer cardiopulmonary bypass time, influencing CS-AKI severity per KDIGO classification; and 3) postoperative prognosis showed no difference in the rate of RRT between the DS and non-DS groups, with no deaths within 14 days after surgery; however, ICU stays were longer in the DS group than in the non-DS group, indicating that upper airway stenosis contributed to the difference.

Incidence of CS-AKI in the DS group

Van den Eynde et al. [4] conducted a meta-analysis on pediatric CS-AKI, reporting a pooled estimated incidence rate of 34.3%. In age-matched cohorts (3 months to 2 years), the CS-AKI incidence averaging at 46.3% and ranged from 12.3% to 70.4% [4]. The analysis showed a higher incidence of CS-AKI in younger age groups, which was consistent with the findings of our study. Neonates and infants have an increased risk of developing AKI due to immature kidneys, and predisposition to ischemia and coagulation, making them particularly susceptible to inflammatory and ischemic changes [4]. Additionally, prolonged cardiopulmonary bypass (CPB) during cardiac surgery can further exacerbate these risks by activating coagulation pathways, resulting in ischemia-reperfusion injury, and reduced GFR, amplifying the effects in younger patients [4]. In our cohort, there was no difference in the frequency of CS-AKI between the DS and non-DS groups; however, these factors likely contributed to the high overall incidence of CS-AKI observed.

We compared body size, length of kidney, renal function, and preoperative complications between children with and without DS requiring surgery for CHD during infancy, typically performed at similar ages in the DS group with CHD. The DS group showed significantly smaller kidney sizes, higher preoperative Cr levels, and lower Cr-eGFR values, suggesting potential renal dysfunction compared to the non-DS group. In a Japanese cohort, renal function in children with DS, assessed by eGFR based on Cr and cystatin C levels, was found to be approximately 80% of the GFR in healthy Japanese children [9]. This is similar to our findings in patients with DS complicated by CHD, suggesting smaller kidneys or fewer nephrons in children with DS [9]. Initially, we anticipated a higher risk of CS-AKI in the DS group due to their smaller kidney size and lower preoperative eGFR.

However, the incidence of CS-AKI was similar between the DS and non-DS groups. Despite having fewer nephrons in hypoplastic kidneys, the remaining healthy nephrons may compensate by increasing GFR and tubular absorption, maintaining normal renal function without a proportionate decline in overall GFR due to renal tissue loss [10]. Furthermore, most patients with DS accompanied by CHD undergo biventricular repair procedures, and complex cardiac diseases such as having a single ventricle is very rare [11]. Children requiring single ventricle repair via the Norwood and Fontan procedures tend to be at higher risk for postoperative AKI due to the complexity of the procedures, prolonged CPB durations, and unstable hemodynamics associated with this physiology [12].

The risk of AKI is higher with single ventricle repair than with biventricular repair, especially in children aged <2 years [12]. Such risk is associated with longer CPB and aortic cross-clamp durations, which are associated with more severe AKI in patients with a single ventricle and often have a negative impact on long-term renal function [12]. In patients with DS, the infrequency of surgeries or the extended duration of surgeries required for single-ventricle circulation or complex congenital heart defects may have also influenced the results of this study. In addition, there was no difference in the number of patients using diuretics and ACE inhibitors preoperatively between the DS and non-DS groups, and no association with AKI was noted in either group. In previous reports, diuretics and ACE inhibitors were not observed to significantly contribute to the risk of developing AKI, possibly due to children having fewer complications and higher renal functional reserve, making them less susceptible to the renal side effects of these drugs [13].

Appropriate estimation of renal function is crucial for pediatric patients undergoing cardiac surgery, particularly those at high risk for CS-AKI. Estimating eGFR in the ICU can be challenging due to rapid renal function changes and delayed sCr level adjustments [14]. However, eGFR remains essential for managing postoperative central venous pressure and fluid balance. Regarding fluid balance, the postoperative fluid volume was lower in the DS group than in the non-DS group. Although fluid management is performed in the ICU after cardiac surgery to prevent excessive fluid balance [15], the DS group had a lower eGFR than the non-DS group, indicating a need for more careful management to avoid more excessive fluid volume. This may have contributed to the prevention of CS-AKI in the DS group.

Serial evaluation of renal function, including eGFR, can enhance a clinician’s ability to assess renal function and may contribute to CS-AKI prevention by facilitating meticulous perioperative fluid management [16,17]. In addition, bacteremia is a significant factor that increases the risk of CS-AKI in children. Studies have shown that bacteremia induces an inflammatory response and reduces blood flow to the kidneys, thereby promoting the development of AKI [4]. Particularly, surgeries involving CPB have a higher risk of bacteremia, which has been shown to increase the incidence of AKI [4]. In our cohort, there was no difference in the incidence of bacteremia between the DS and non-DS groups, and there were no cases that led to severe AKI or death. Appropriate antibiotic treatment and early infection management were considered crucial for preventing postoperative AKI.

Risk factors for CS-AKI in the DS group

This study revealed that younger age and longer cardiopulmonary bypass time were risk factors for post-cardiac surgery CS-AKI in the entire cohort and DS groups. This finding may be attributed to the inherent immaturity of kidneys and renal tubules in children under 2 years of age, as the maximum glomerular filtration rate is not achieved until this age. In younger patients, prolonged cardiopulmonary bypass can activate the coagulation system, leading to ischemia-reperfusion injury, systemic inflammation, and decreased GFR, resulting in reduced resilience [18-22]. Furthermore, KDIGO criteria for classifying the severity of CS-AKI have been demonstrated to be useful even after CHD surgery in infants [23]. Therefore, it is important to consider these factors in the perioperative management of patients with DS with potential renal dysfunction after cardiac surgery.

Postoperative prognosis in CS-AKI in patients with DS

In our cohort, three patients (2.1%) were diagnosed with severe AKI (KDIGO 3) in the early postoperative period and required continuous RRT, which did not differ between the DS and non-DS groups but was essential for ICU management in all patients. Severe AKI can lead to left ventricular dysfunction due to cardiorenal syndrome, activating the sympathetic nervous system and the renin-angiotensin-aldosterone system, causing vasoconstriction and disturbances in acid-base and coagulation balances, creating a vicious cycle of further damage [23]. In this context, RRT helps maintain fluid and electrolyte balance, removes metabolic waste products, toxins, and inflammatory mediators from blood, and provides a stable hemodynamic profile [24]. Although there is no consensus on the optimal timing of RRT initiation, we believe that, despite the small sample size of our cohort, its use contributed to improved outcomes in cases of severe AKI.

To detect CS-AKI at an early stage, traditional biomarkers such as sCr levels and urine output have important limitations. Stress markers, such as tissue inhibitor of metalloproteinases-2 and insulin-like growth factor binding protein 7, as well as damage markers like the neutrophil gelatinase-associated lipocalin, kidney injury molecule-1, and liver fatty acid-binding protein, have enabled a more precise delineation of the etiology, pathophysiology, site, mechanisms, and severity of injury, enabling early diagnosis and prognostication of AKI [25]. Validation of these markers is desirable in the context of pediatric cardiac surgery-associated AKI. Understanding these risk factors for CS-AKI severity and evaluating renal function during perioperative management can enhance patient outcomes.

Although no deaths occurred during the perioperative period in our study, airway obstruction disorders were identified as factors contributing to prolonged ICU stays in the DS group after CHD surgery. DS is associated with a high incidence of airway obstruction disorders, with studies reporting rates of upper airway narrowing, such as laryngomalacia, in 43% of cases [26]. Patients with DS and concurrent airway obstruction disorders often have heart disease with pulmonary hypertension, necessitating tracheostomy or facing postoperative mortality [27]. Airway obstruction disorders often complicate extubation difficulties and may worsen due to surgical invasiveness, secondary inflammation from endotracheal intubation and mechanical ventilation, postoperative infections, and cardiomegaly [28]. Given the increased risk associated with upper airway narrowing, careful perioperative management is essential in patients with DS and airway obstruction disorders.

Study limitations

This study had some limitations. Firstly, this study was conducted with a relatively small sample size at a single institution, potentially limiting the generalizability of the findings. Secondly, the study lacked assessments such as kidney volume measurement via ultrasound and pathological tissue evaluation, which could provide deeper insights into renal function. Thirdly, the absence of evaluations like inulin clearance or 24-hour Cr clearance measurements may have impacted the comprehensive understanding of renal function. Fourthly, as all participants were Japanese, one of the healthiest and equal countries in the world, resulting in less pronounced social inequality. In contrast, in regions with low socioeconomic status (SES), people face systemic barriers related to low SES, which can adversely affect cardiovascular health [2], complicating life, creating health disparities, and causing social difficulties for patients with DS.

We hope that the findings of this study will contribute to interventions that address the needs of diverse communities and improve the overall prognosis for patients with DS with CHD. Finally, the heterogeneous nature of the CHD population could affect the generalization of the findings regarding the impact of CS-AKI within this group.

## Conclusions

In patients with DS and CHD, smaller kidney sizes and lower preoperative Cr-eGFR levels were observed compared to patients without DS having CHD. However, no disparity was found in the incidence or severity of CS-AKI. Moreover, younger age at the time of surgery and longer cardiopulmonary bypass time were strongly associated with the development and severity of CS-AKI in the DS group with CHD, as per the KDIGO criteria. Additionally, prolonged ICU stays were linked to upper airway obstruction disorders. Understanding and managing potential renal dysfunction in patients with DS and CHD may help prevent perioperative AKI after cardiac surgery.
